# Predictive utility of fibrinogen in acute kidney injury in living donor liver transplantation: A propensity score-matching analysis

**DOI:** 10.1371/journal.pone.0252715

**Published:** 2021-06-04

**Authors:** Jaesik Park, Min A. Joo, Ho Joong Choi, Sang Hyun Hong, Chul Soo Park, Jong Ho Choi, Min Suk Chae

**Affiliations:** 1 Department of Anesthesiology and Pain Medicine, Seoul St. Mary’s Hospital, College of Medicine, The Catholic University of Korea, Seoul, Republic of Korea; 2 Department of Surgery, Seoul St. Mary’s Hospital, College of Medicine, The Catholic University of Korea, Seoul, Republic of Korea; Ohio State University Wexner Medical Center Department of Surgery, UNITED STATES

## Abstract

**Background:**

This study investigated the association between the fibrinogen level and the risk of acute kidney injury (AKI) in patients who have undergone living donor liver transplantation (LDLT).

**Patients and methods:**

A total of 676 patients who underwent LDLT were analyzed retrospectively. Exclusion criteria included a history of severe kidney dysfunction, emergency operation, deceased donor, ABO-incompatible transplantation, and missing data. The study population was divided into low and normal fibrinogen groups. A 1:1 propensity score (PS) matching analysis was used to evaluate the association between a low fibrinogen level (< 160 mg/dL) and postoperative development of AKI.

**Results:**

In total, 142 patients (23.1%) developed AKI after LDLT. The PS matching analysis showed that the probability of AKI was two-fold higher in the low fibrinogen group than in the normal fibrinogen group. In addition, patients with AKI had poorer postoperative outcomes such as longer hospitalization, longer ICU stay, and higher mortality than patients without AKI.

**Conclusions:**

The preoperative fibrinogen level may be useful for risk stratification of patients undergoing LDLT in terms postoperative development of AKI.

## Introduction

Living donor liver transplantation (LDLT) is a widely performed operation for patients with end-stage liver disease [1]. However, various postoperative complications can occur, and mortality is higher in such patients [2] Therefore, early identification of relevant risk factors and proper management are crucial for a good prognosis. Acute kidney injury (AKI) is a common complication of LDLT, affecting about 30% of patients [3, 4]. Various factors are related to the development of AKI after liver transplantation (LT), including the Model for End-Stage Liver Disease (MELD) score, chronic kidney disease (CKD), diabetes mellitus (DM), body mass index (BMI), and donor age [4–7]. Preoperative systemic inflammation may also increase the possibility of postoperative AKI. Inflammatory markers, including C-reactive protein (CRP) and albumin, have been associated with the development of AKI [8]. Preoperative levels of cytokines, including interleukin (IL)-6 and IL-10, are associated with the development of postoperative AKI after major surgery, such as cardiac or liver transplant surgery [9, 10].

Fibrinogen is a glycoprotein known for its crucial role in hemostasis [11]. However, fibrinogen is also related to other medical conditions, including systemic inflammation and AKI [12–14]. In those reports, fibrinogen was associated with the development of AKI after major surgery, such as cardiac or abdominal aortic aneurysm repair surgery. As AKI increases the risk of postoperative complications, such as infection, early graft dysfunction, graft failure, or death [15, 16], risk factors for AKI, including low fibrinogen, should be identified, particularly in high-risk patients undergoing LDLT.

We investigated the relationship between low fibrinogen level and the development of AKI after LDLT. We compared postoperative outcomes between patients in AKI and non-AKI groups, and suggest that fibrinogen is helpful for estimating the risk of AKI in patients who have undergone LDLT.

## Patients and methods

### Ethical considerations

The Institutional Review Board of Seoul St. Mary’s Hospital Ethics Committee (KC20RISI0176) approved this study on April 6, 2020. This study used a retrospective design, so the requirement for informed consent was waived.

### Study population

Laboratory data were retrospectively collected from 676 adult patients who underwent LDLT between January 2009 and December 2019 at Seoul St. Mary’s Hospital. The exclusion criteria included emergency surgery, deceased donor liver transplantation (DDLT), history of kidney dysfunction (CKD [17], hepatorenal syndrome, or history of dialysis), age < 19 years, and missing data. Finally, 51 patients were excluded, and 615 patients were analyzed.

### Living donor liver transplantation

The surgical and anesthetic methods for LDLT have been described in detail previously [18]. In our patients, the piggyback technique was used in the right hepatic lobe to preserve the recipient’s caval vein. The portal vein, hepatic artery, hepatic vein, and bile duct were anastomosed, and the flow and patency of the hepatic artery and portal vein were evaluated by Doppler ultrasonography (Prosound SSD-5000; Hitachi Aloka Medical, Tokyo, Japan). A portocaval shunt, simultaneous splenectomy, and splenic artery ligation were performed to modulate portal flow based on the surgeon’s judgment.

Balanced anesthesia and proper hemodynamic management were applied during surgery. The hemodynamic goals were a mean blood pressure ≥ 65 mmHg, central venous pressure ≤ 10 mmHg, and mean pulmonary artery pressure ≤ 25 mmHg. The bispectral index value was monitored and maintained within the target range (40–60). Blood products were transfused based on existing guidelines [19]. Packed red blood cells (PRBCs) were transfused to maintain the hematocrit level at > 25%, and fresh-frozen plasma (FFP) and single-donor platelet (SDP) transfusion were performed according to the laboratory results and thromboelastography. Severe postreperfusion syndrome (PRS) was classified as follows: decrease in mean blood pressure ≥ 30% or hypotensive duration ≥ 5 min, severe arrhythmias, such as asystole or ventricular tachycardia, use of strong rescue vasopressors (i.e., epinephrine or norepinephrine), and antifibrinolytic treatment due to ongoing fibrinolysis [20].

Immunosuppressant drugs, such as mycophenolate mofetil, calcineurin inhibitor, and prednisolone, were administered and tapered after LDLT based on our hospital’s protocol. In addition, the monoclonal antibody basiliximab was administered before surgery and on postoperative day 4.

### Acute kidney injury

AKI was defined based on the Kidney Disease Improving Global Outcomes criteria, because of the more accurate prognoses achieved with these criteria compared to those of the Risk/Injury/Failure/Loss/End-stage and Acute Kidney Injury Network [21]. AKI was diagnosed as follows: stage 1, increase in serum creatinine (sCr) ≥ 0.3 mg/dL (within 48 hours) or baseline sCr (within the first 7 days) multiplied by 1.5–1.9; stage 2, baseline sCr multiplied by 2.0–2.9; stage 3, increase in sCr ≥ 4.0 mg/dL or baseline sCr multiplied by ≥ 3.0, or beginning renal replacement therapy [22]. The study population was classified into non-AKI and AKI groups.

### Fibrinogen measurements

Fibrinogen and other laboratory samples were collected preoperatively in all patients scheduled for LDLT. Venous or arterial blood samples were collected into Clot Activator Tubes/BD Vacutainers (Becton, Dickinson and Co., Franklin Lakes, NJ, USA) on the day before surgery, and analyzed using an automated chemistry analyzer (Hitachi 7600; Hitachi Ltd., Tokyo, Japan). Fibrinogen samples were collected (Sodium Citrate Tube/ BD Vacutainer; Becton, Dickinson and Co.) on the day before surgery and analyzed using an automated blood coagulation analyzer (CS-5100; Sysmex Corp., Kobe, Japan). A fibrinogen level < 160 mg/dL was defined as low, according to our laboratory range and a previous report [23]. If data were collected several times in a single day, the data collected nearest to the time of the surgery were included in the analysis.

### Perioperative recipient and donor characteristics and findings

Preoperative recipient characteristics and findings of interest included BMI, sex, age, etiology of liver disease, MELD score, comorbidities (hypertension and DM), hepatic decompensation parameters (encephalopathy [West-Haven grade I–II] [24], ascites, and varix), transthoracic echocardiography parameters (ejection fraction, diastolic dysfunction, left ventricular mass index, and cardiomyopathy [25]), and laboratory data (sodium, potassium, calcium, glucose, white blood cell [WBC] count, platelet count, albumin, sCr, and ammonia). Intraoperative recipient findings included vital signs (mean heart rate, mean blood pressure, and central venous pressure), mean lactate, PRS [26], numbers of transfused blood products (PRBCs, FFP, and SDPs), surgical duration, hourly fluid infusion, and urine output. Donor characteristics and findings of interest included age, sex, graft-recipient weight ratio, graft ischemic time, and donor graft fatty change. Postoperative findings in recipients included nephrotoxic drug (Lasix [furosemide]) administration, the trough level of calcineurin inhibitors, and mean blood pressure to estimate the perfusion pressure. Postoperative outcomes included length of hospital and intensive care unit (ICU) stays and overall patient mortality.

### Statistical analysis

A 1:1 propensity score (PS) matching analysis without replacement was used to adjust for confounders in the low and normal fibrinogen groups [27]. We compared perioperative factors before and after matching using the Mann–Whitney *U* test and the chi-square test or Fisher’s exact test, as appropriate. The relationship between low fibrinogen (< 160 mg/dL) and postoperative AKI was analyzed using the chi-square test and multivariate logistic regression with PS adjustment. Continuous data are expressed as the interquartile range (IQR) and median, and categorical data are expressed as numbers and proportions. To determine the incidence of AKI according to the postoperative day, Spearman’s correlation analysis was performed. SPSS for Windows (*ver*. 24.0; SPSS Inc., Chicago, IL, USA) and MedCalc for Windows software (*ver*. 11.0; MedCalc Software, Ostend, Belgium) were used to conduct the statistical analyses.

## Results

### Demographic characteristics of the patients undergoing LDLT

The study population was predominantly male (69.9%) and the median (IQR) age, BMI, MELD score, ejection fraction, and fibrinogen level were 54 (48–59) years, 24.2 (22.1–26.6) kg/m^2^, 13.6 (6.6–23.7) points, 64.5% (62–67%), and 161 (112–218) mg/dL, respectively. The etiologies of LDLT were hepatitis B virus (HBV) (56.4%), alcoholic hepatitis (19.7%), hepatitis C (7.3%), autoimmune hepatitis (4.2%), hepatitis A (4.2%), drug and toxic hepatitis (2.0%), and cryptogenic hepatitis (6.2%). The prevalence rates of diabetes, hypertension, encephalopathy, ascites, and varix were 26.3% (n = 162), 20.2% (n = 124), 34.1% (n = 210), 47% (n = 289), and 24.2% (n = 149), respectively. The incidence of AKI after the surgery was 23.1% (n = 142).

### Perioperative recipient and donor-graft factors before and after PS matching

Before PS matching, significant differences were observed between the low and normal fibrinogen groups in preoperative findings (i.e., MELD score, encephalopathy, ascites, hemoglobin, albumin, platelet count, sodium, total bilirubin, ammonia, international normalized ratio [INR]) and intraoperative findings (i.e., PRC, FFP, platelet concentrate, and hourly urine output) (Table 1). After PS matching, no significant differences were observed between the groups and the absolute standardized differences were < 0.25.

**Table 1:** Comparison of perioperative findings between the low and normal fibrinogen groups using propensity score matching analysis.

	Before propensity score matched analysis				After propensity score matched analysis			
Group	Low Fibrinogen	Normal Fibrinogen	*P*	SD	Low Fibrinogen	Normal Fibrinogen	*p*	SD
**n**	**304**	**311**			**168**	**168**		
***Preoperative finding***								
Age (years)	52 (47–58)	55 (49–60)	0.002	-0.202	53 (47–60)	54 (47–59)	0.995	0.032
Sex (male)	201 (66.1%)	229 (73.6%)	0.042	-0.159	113 (67.3%)	111 (66.1%)	0.817	-0.025
Body mass index (kg/m^2^)	24.6 (22.3–27)	23.8 (21.9–26.1)	0.004	0.233	24.2 (22.1–27.0)	24.2 (22.1–26.5)	0.239	0.036
Diabetes	75 (24.7%)	87 (28.0%)	0.352	-0.076	41 (24.4%)	46 (27.4%)	0.533	-0.069
Hypertension	44 (14.5%)	80 (25.7%)	0.001	-0.319	31 (18.5%)	32 (19.0%)	0.889	-0.017
MELD score (points)	18.05 (9.9–27.0)	9.8 (4.9–18.5)	<0.001	0.597	15.4 (8.8–24.2)	13.8 (7.5–24.1)	0.545	0.050
Encephalopathy	123 (40.5%)	87 (28.0%)	0.001	0.254	58 (34.5%)	57 (33.9%)	0.908	0.012
Varix	86 (28.3%)	63 (20.3%)	0.020	0.178	41 (24.4%)	40 (23.8%)	0.899	0.013
Ascites	173 (56.9%)	116 (37.3%)	<0.001	0.395	96 (57.1%)	87 (51.8%)	0.324	0.108
LVMI (g/m^2^)	90.6 (79.1–98.3)	87.9 (75–99.4)	0.020	0.026	91.1 (79.7–100.6)	87.4 (74.2–100.0)	0.144	0.058
Cardiomyopathy	35 (11.5%)	39 (12.5%)	0.695	-0.032	21 (12.5%)	23 (13.7%)	0.747	-0.037
Ejection Fraction	64.5 (62–67)	64.5 (62–67)	0.495	0.055	64.5 (61–67)	64.5 (62.7–67)	0.839	-0.038
Diastolic Dysfunction	128 (42.1%)	130 (41.8%)	0.939	0.006	70 (41.7%)	69 (41.1%)	0.912	0.012
*Laboratory variables*								
Hemoglobin (g/dL)	9.2 (7.9–10.9)	10.2 (8.7–12.4)	<0.001	-0.446	9.5 (8.2–11.2)	9.3 (8.3–11.2)	0.911	0.014
White blood cell count (x 10^9^/L)	4.8 (2.8–8.9)	4.1 (2.9–5.7)	0.005	0.191	4.5 (2.8–8.0)	3.7 (2.5–5.8)	0.076	-0.021
Albumin (g/dL)	2.9 (2.6–3.3)	3.2 (2.7–3.7)	<0.001	-0.514	3.0 (2.7–3.4)	2.9 (2.6–3.4)	0.497	0.084
Platelet count (x 10^9^/L)	52.5 (39–75)	84 (56–129)	<0.001	-0.779	57.5 (40–83.5)	62 (48–92.5)	0.047	-0.068
Sodium (mEq/L)	138 (134–141)	140 (137–142)	<0.001	-0.286	139 (135–141)	139 (136–142)	0.394	-0.050
Potassium (mEq/L)	4 (3.6–4.3)	4 (3.7–4.3)	0.820	-0.016	4.0 (3.6–4.4)	4.0 (3.6–4.4)	0.591	0.001
Calcium (mEq/L)	8.4 (7.9–8.9)	8.4 (8.0–8.8)	0.380	0.021	8.4 (8.0–8.9)	8.3 (7.9–8.8)	0.183	0.119
Glucose (mg/dL)	113 (93.0–148.8)	105 (91–129)	0.033	0.164	109 (92–141)	106 (90–131)	0.488	0.048
Urea Nitrogen	16.8 (10.5–26.5)	14.8 (11.7–23)	0.143	0.069	16 (11–26)	15 (12–27)	0.832	0.034
Creatinine (mg/dL)	0.81 (0.62–1.29)	0.88 (0.69–1.08)	0.210	0.027	0.78 (0.62–1.20)	0.88 (0.69–1.29)	0.049	-0.038
Total Bilirubin	5.1 (1.8–18.7)	1.2 (0.7–4.4)	<0.001	0.423	3.3 (1.3–17.5)	2.2 (1.0–14.4)	0.182	0.082
Lactate (mg/dL)	5.2 (3.8–6.5)	5.0 (3.7–6.4)	0.567	0.064	4.9 (3.9–6.4)	5.1 (3.8–6.5)	0.853	-0.094
Ammonia	106.5 (71–170)	90 (61–139)	0.001	0.251	104 (71–167)	105 (64–160)	0.596	0.006
INR	1.8 (1.4–2.3)	1.3 (1.1–1.6)	<0.001	0.598	1.6 (1.3–2.2)	1.5 (1.3–1.9)	0.031	0.121
***Intraoperative finding***								
Total surgery duration (min)	512.5 (455–592.5)	510 (460–570)	0.929	-0.024	500 (445–560)	508 (465–580)	0.227	-0.066
Severe PRS (Class > 1)	164 (53.9%)	163 (49.8%)	0.703	0.031	87 (51.8%)	83 (49.4%)	0.662	0.048
*Average of vital signs*								
MBP (mmHg)	73.4 (67.3–79.1)	74.7 (69.0–80.7)	0.039	-0.183	74.1 (67.2–79.2)	74.2 (67.2–79.2)	0.497	-0.094
HR (beats/min)	90 (78.5–100.9)	90 (80.5–98.3)	0.763	-0.036	90.3 (77.6–100.1)	89.4 (80.1–98.7)	0.818	-0.032
CVP (mmHg)	9.3 (7.5–11.5)	9.0 (7.0–10.8)	0.027	0.179	9.3 (7.5–11.3)	9.1 (7.3–11.0)	0.526	0.053
*Blood product transfusion (unit)*								
Packed red blood cell	10 (6–14)	5 (3–12)	<0.001	0.297	9 (5–15)	8 (3–13)	0.061	0.130
Fresh frozen plasma	9 (6–13)	5 (4–10)	<0.001	0.423	8 (5–12)	6 (4–10)	0.018	0.072
Platelet concentrate	5 (0–10)	0 (0–5)	<0.001	0.334	5 (0–10)	5 (0–10)	0.300	-0.012
Hourly fluid infusion (mL/kg/h)	10.6 (8.0–14.2)	10.6 (8.2–14.4)	0.602	0.063	10.8 (8.3–14.6)	10.5 (7.9–14.5)	0.864	0.078
Hourly urine output (mL/kg/h)	0.99 (0.52–1.81)	1.5 (0.83–2.3)	<0.001	-0.301	1.02(0.58–1.86)	1.15 (0.53–2.08)	0.313	-0.036
***Postoperative finding***								
Nephrotoxic drug exposure (Lasix)	32 (10.5%)	31 (10.0%)	0.819	0.018	15 (8.9%)	18 (10.7%)	0.582	-0.058
Lasix dose (mg)	20 (20–20)	20 (20–20)	0.781	0.055	20 (20–20)	20 (20–20)	0.533	-0.101
Calcineurin inhibitor level (ng/ml)	7.8 (6.6–9.4)	7.6 (6.6–9.1)	0.260	0.114	7.5 (6.4–9.2)	7.7 (6.8–9.4)	0.522	-0.065
Mean blood pressure (mmHg)	76 (69–84)	77 (69–86)	0.184	0.013	76 (70–85)	76 (67–85)	0.911	-0.094
***Donor-graft finding***								
Age (years)	35 (26–42)	35 (26–41)	0.718	0.020	35 (26–40)	35 (25–43)	0.901	-0.040
Sex(male)	183 (60.2%)	195 (62.7%)	0.524	0.051	101 (60.1%)	100 (59.5%)	0.911	-0.012
GRWR (%)	1.24 (0.99–1.48)	1.23 (1.07–1.54)	0.260	-0.092	1.27 (1.01–1.50)	1.22 (1.03–1.48)	0.731	0.026
Graft ischemic time (min)	109 (80.3–111.0)	105 (71–109)	0.003	0.207	109 (78–109)	109 (72–109)	0.488	0.084
Fatty change (%)	1 (0–5)	2 (0–5)	0.223	-0.080	2 (0–5),	2.5 (0–5)	0.599	0.058

**Abbreviations:** MELD, model for end-stage liver disease; LVMI, left ventricular mass index; INR, international normalized ratio; PRS, post reperfusion syndrome; MBP, mean blood pressure; HR, heart rate; CVP, central venous pressure; GRWR, graft recipient weight ratio

**NOTE:** Values are expressed as median (interquartile) and numbers (proportions).

### Timing of AKI development

The incidence of postoperative AKI was higher closer to the day of surgery (*p* = 0.005) (S1 Table).

### Incidence of postoperative AKI in PS-matched patients by fibrinogen level

The incidence of postoperative AKI was higher in patients with a low fibrinogen level than in those with a normal fibrinogen level (Table 2).

**Table 2:** The incidence of postoperative AKI according to fibrinogen level.

Group	Low fibrinogen (<160 mg/dL)	Normal fibrinogen (160–400 mg/dL)	*p*
**n**	**168**	**168**	0.004
Absence of AKI	119 (70.8%)	141 (83.9%)	
Presence of AKI	49 (29.2%)	27 (16.1%)	

**Abbreviations:** AKI, acute kidney injury; PS, propensity score

**NOTE:** Values are expressed as numbers (proportions).

### Fibrinogen level according to the development of AKI in PS-matched patients

**Fig 1:**
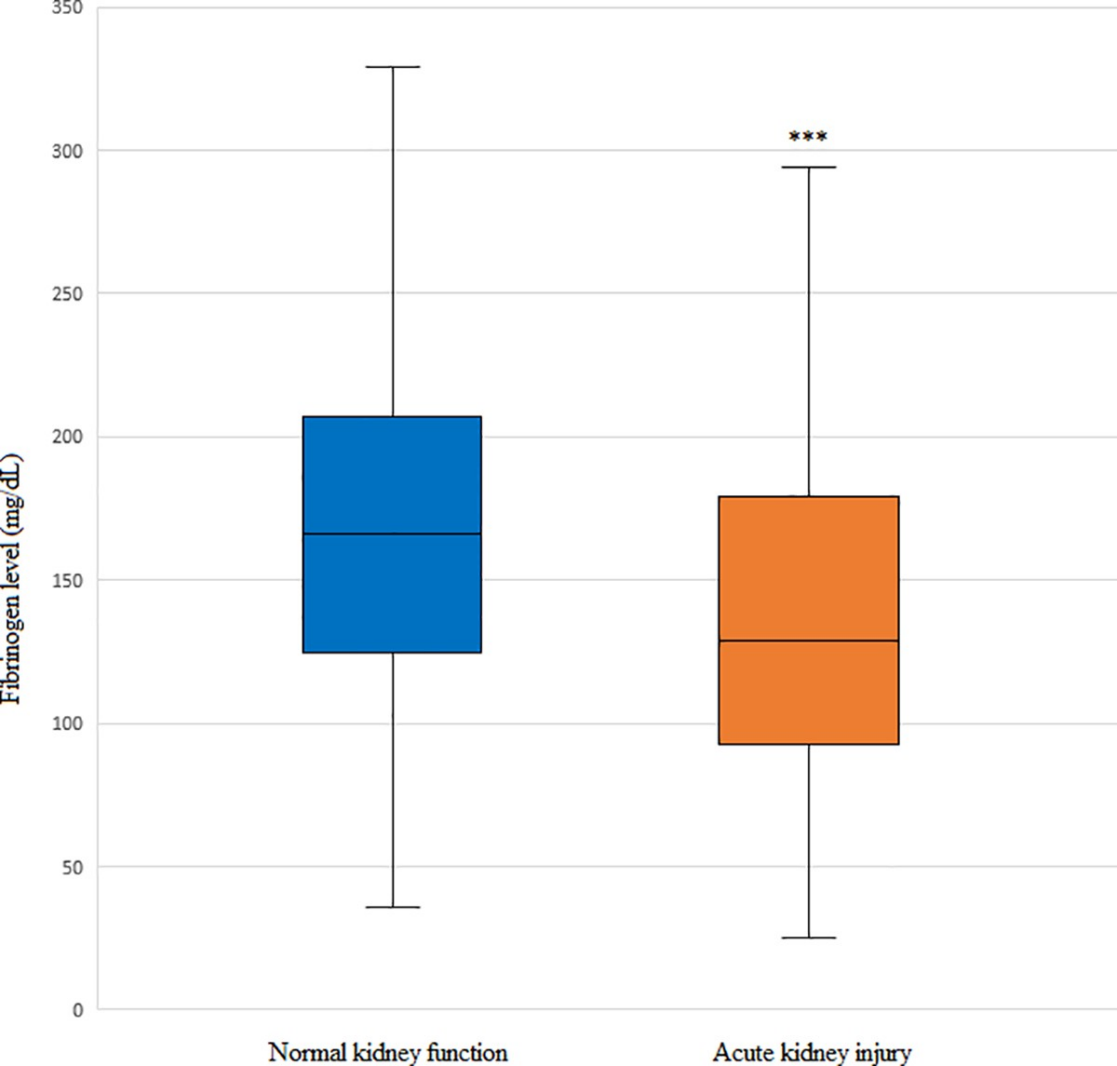
Comparison of serum fibrinogen levels according to the development of acute kidney injury (AKI) in patients who underwent living donor liver transplantation (LDLT). The box plots show the median (line in the middle of the box), interquartile range (box), and 5^th^ and 95^th^ percentiles (whiskers). ***p < 0.001 vs. normal kidney function.

Patients in the AKI group showed lower median and IQR fibrinogen levels (Fig 1). The median (IQR) levels of fibrinogen were 128.5 (92.3–179) mg/dL and 166 (123.5–208.3) mg/dL in patients with and without AKI, respectively.

### Fibrinogen level according to the AKI stage in PS-matched patients

**Fig 2:**
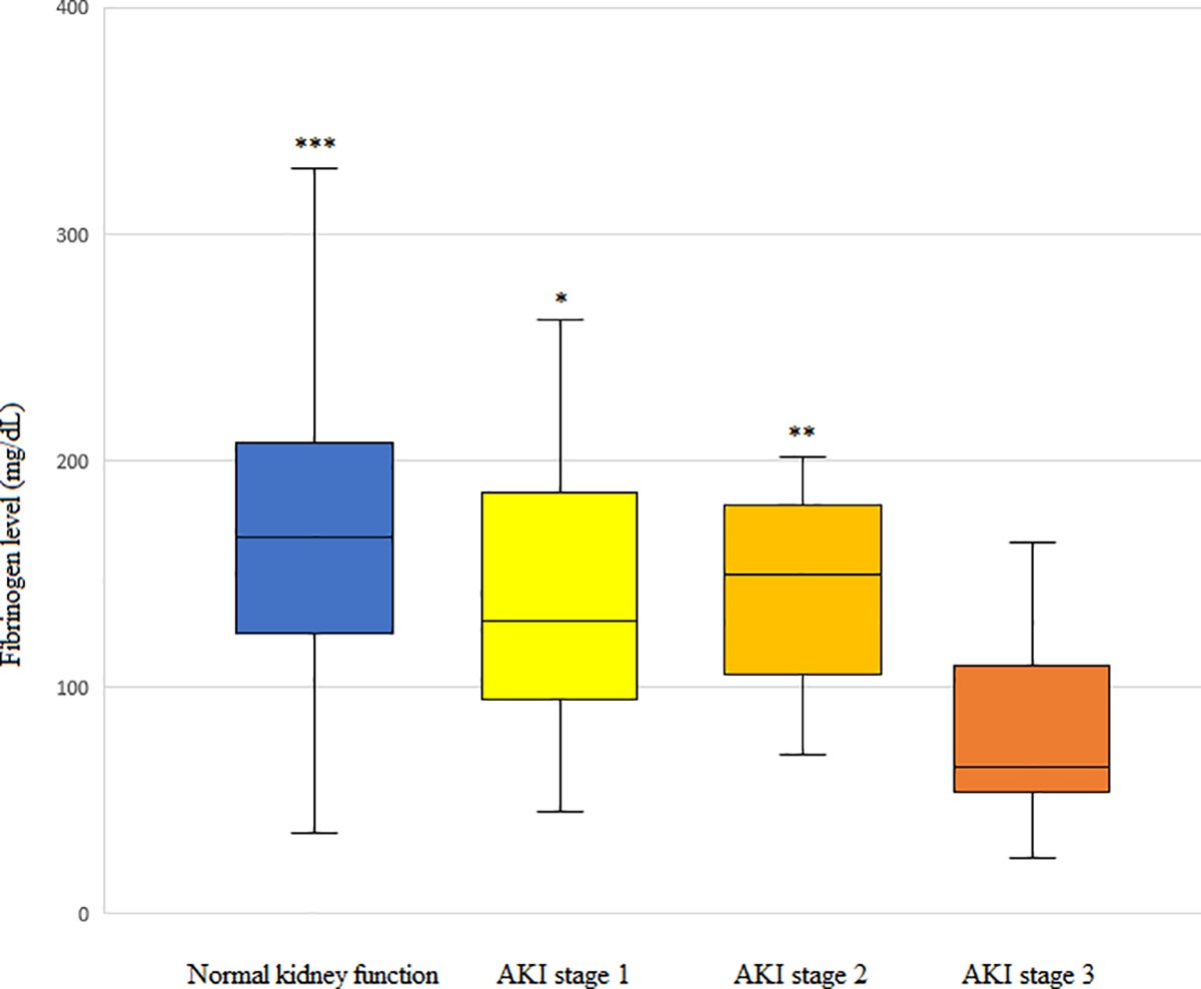
The preoperative fibrinogen level according to the severity of acute kidney injury (AKI). The box plots show the median (line in the middle of the box), interquartile range (box), and 5^th^ and 95^th^ percentiles (whiskers). *p = 0.013 vs. AKI stage 3, **p = 0.006 vs. AKI stage 3, *** *p* = 0.001 vs. AKI stage 3.

Patients with severe AKI (AKI stage 3) had significantly lower preoperative fibrinogen levels than those with AKI stage 1 or 2 (Fig 2).

### Association between low fibrinogen level and postoperative development of AKI in PS-matched patients

A low fibrinogen level was associated with the development of AKI, both in the entire study population and in the PS-matched patients (Table 3). A low fibrinogen level remained a factor related to the development of AKI after PS adjustment (*p* = 0.005).

**Table 3:** Association between low fibrinogen level and postoperative AKI.

	*ß*	Odds ratio	95% CI	*p*
**In the entire study population (n = 615)**				
Low fibrinogen (vs. normal fibrinogen)† adjusted for PS	0.897	2.453	1.657–3.630	<0.001
**In the PS-matched study population (n = 336)**				
Low fibrinogen (vs. normal fibrinogen)† adjusted for PS	0.766	2.150	1.267–3.651	0.005

**Abbreviations:** AKI, acute kidney injury; PS, propensity score

^†^(*vs*. reference values)

### Association between the fibrinogen level and postoperative development of AKI after adjusting for preoperative factors

A low fibrinogen level was associated with the development of AKI in the entire study population after adjusting for preoperative factors, including the MELD score, diabetes mellitus, BMI, albumin level, and PS. After PS matching, a low fibrinogen level was still associated with the development of AKI after adjusting for those preoperative factors and PS (S2 Table).

### Comparison of preoperative findings between the low and normal PS-matched groups

**Fig 3:**
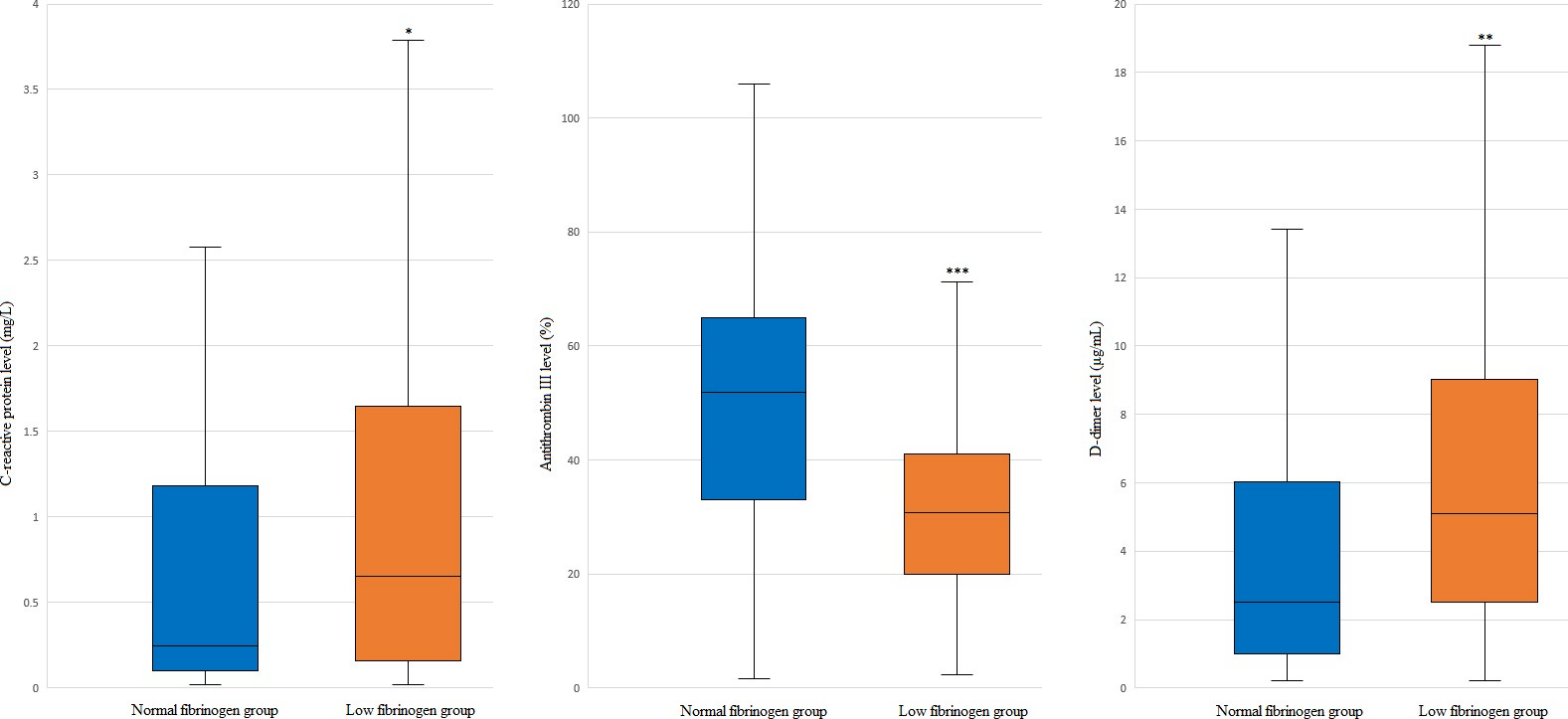
Comparison of preoperative findings between patients in the low and normal fibrinogen groups who underwent living donor liver transplantation (LDLT). The box plots show the median (line in the middle of the box), interquartile range (box), and 5^th^ and 95^th^ percentiles (whiskers). *p = 0.004 vs. normal fibrinogen, **p = 0.001 vs. normal fibrinogen, *** *p* < 0.001 vs. normal fibrinogen.

Patients in the low fibrinogen group had higher CRP levels (*p* = 0.004). In addition, the antithrombin III and d-dimer levels showed significant differences between the groups (*p* = < 0.001 and 0.001, respectively, Fig 3).

### Postoperative complications in PS-matched patients

The PS-matched patients in the AKI group had a longer duration of hospitalization, longer duration of ICU stay, and higher overall mortality rate than those without AKI (*p* = 0.015, 0.02, and 0.036, respectively).

## Discussion

The main finding of our study was that low fibrinogen level was associated with the risk of developing AKI after LDLT. In the PS-matched patients, a low fibrinogen level (< 160 mg/dL) was associated with the risk of developing AKI. The risk was about twofold higher in patients with a low fibrinogen level than in those with a normal fibrinogen level.

Postoperative AKI is a relatively common, and serious, complication of LT, occurring in 20–30% of recipients [3–5, 28–30]. Rhaman *et al*. [28] reported that hepatic ischemia-reperfusion injury and postoperative AKI were related to poorer survival. Thongprayoon *et al*. [29] reported that patients with AKI after LT have a higher risk of liver graft failure. In the present PS matching analysis, patients with AKI had more postoperative complications, such as longer hospital and ICU stays, and a higher mortality rate than patients with normal kidney function. Risk factors for postoperative AKI include reduced renal perfusion, inflammation, and toxins [31–33]. Systemic inflammation is an important factor in the development of AKI [34]. Higher levels of CRP, WBCs, and inflammatory markers are associated with the development of AKI due to inhibition of G1/S-dependent tubular epithelial cell regeneration [35, 36]. Inflammatory mediators, such as IL-6 and tumor necrosis factor-alpha, affect tubular epithelial cells, resulting in loss of function [37].

Fibrinogen is a soluble glycoprotein that plays a critical role in hemostasis and coagulation, as a substrate for fibrin and a facilitator of platelet aggregation [11, 38]. Fibrinogen is produced in the liver and its blood concentration is maintained at 160–400 mg/dL [23]. In addition to its role in hemostasis, fibrinogen is also implicated in inflammation, tissue injury, and bacterial infection [12]. Fibrinogen plays a proinflammatory role and is related to many clinical conditions, such as vascular wall disease [39]. Fibrinogen has been associated with the development of AKI in previous studies. A high preoperative fibrinogen level in patients undergoing cardiac surgery is a risk factor for postoperative development of AKI [13]. Celik *et al*. [14] reported that elevated serum fibrinogen was associated with the occurrence of contrast-induced AKI in patients undergoing percutaneous coronary intervention. These studies suggested that inflammation may be the main cause of AKI. Hoffmann *et al*. [40] reported that fibrinogen is an early diagnostic biomarker of AKI in patients undergoing abdominal aortic aneurysm repair.

No study has investigated the relationship between fibrinogen level and the development of AKI in patients with liver cirrhosis. In addition, the association between preoperative fibrinogen level and postoperative AKI has not been studied. Serum fibrinogen levels often decrease due to impaired hepatic function in patients with hepatitis or cirrhosis [41–43]. Shah *et al*. [43] reported that 40% of patients with cirrhosis had low fibrinogen levels. A low fibrinogen level is related to higher mortality in patients with acute-on-chronic hepatitis B liver failure [41]. Arif *et al*. [44] reported that fibrinogen levels increased in patients with mild-to-moderate cirrhosis. However, fibrinogen levels decreased in patients with severe cirrhosis. Consistent with this, patients in the present study with low fibrinogen levels seemed to be more vulnerable to AKI. As fibrinogen is generated in functional hepatocytes, a low fibrinogen level could be a surrogate marker for hepatic decompensation, which could increase the possibility of the development of AKI in cirrhotic patients [45]. Therefore, unlike patients in previous studies in which AKI was related to high fibrinogen levels and inflammation [13, 14], in patients undergoing LDLT, low fibrinogen levels could be a risk factor for AKI.

Fibrinogen is mainly produced in the liver and its level may be a useful indicator of the synthetic capability of the liver. Exacerbation of liver dysfunction may be related to an inappropriate response to inflammation and inefficient replacement of consumed fibrinogen, which results in a decrease in the level thereof [46]. In previous studies, patients with severe liver decompensation showed lower levels of fibrinogen than those with mild-to-moderate liver decompensation [44]. However, the synthetic ability of the liver cannot be assessed directly based on a single factor, such as fibrinogen. Factor V is produced mainly by the liver, and its level decreases during hepatic failure, whereas factor VIII increases during hepatic failure due to extrahepatocyte synthesis [46]. As these factors play roles in controlling the anti-coagulation cascade along with fibrinogen, the levels of these factors may increase the accuracy of hepatic decompensation, which affects kidney injury together with the fibrinogen level. In this study, the low fibrinogen group had lower antithrombin III and higher d-dimer levels. These patients were more likely to develop AKI, consistent with the results of previous studies in which the antithrombin level was lower, and the d-dimer level higher, in the AKI than non-AKI group [47, 48]. Because the activation of coagulation and fibrinolysis is associated with hepatic decompensation [49, 50], a more advanced disease status could be the cause of AKI [45].

The MELD score is calculated based on sCr, the INR, and serum bilirubin, and the severity of hepatic function is determined using the MELD score (> 25 points, severe decompensation; ≤ 25 points, mild-to-moderate decompensation) [51]. Cirrhotic patients undergoing LDLT have a better hepatic condition than those undergoing DDLT (mean MELD score = 15 ± 6 points vs. 24 ± 7 points, respectively) [52]. Our patients who underwent LDLT had tolerable hepatic function (mean MELD score = 15.7 ± 11 points); among these patients, those with fibrinogen level within the normal range seemed to be less susceptible to AKI after surgery than those with subnormal fibrinogen levels. Therefore, low fibrinogen level may be an early and easily measurable surrogate marker for hepatic dysfunction that subsequently increases the risk of postoperative AKI in patients scheduled for LDLT.

Some limitations of the present study should be discussed. First, we were unable to identify the mechanisms underlying the association between low fibrinogen level and the development of AKI. Although the factors related to hepatic decompensation were not significantly different between the two groups after PS matching, the hepatic synthetic function of inflammatory proteins, including fibrinogen, may be lower in the low fibrinogen group than the normal fibrinogen group. Therefore, we suggest that a low fibrinogen level is an early marker of hepatic decompensation, a more advanced disease status, and a risk of AKI development. Inflammation, coagulation, and fibrinolysis were associated with the development of AKI. However, it is difficult to determine whether those processes were responsible for the development of AKI, or whether coexisting hepatic decompensation was the mechanism; further studies are required. Second, despite the PS matching analysis, biases could have remained due to the retrospective study design. Third, in previous studies, patients who underwent DDLT developed postoperative AKI more frequently than those who underwent LDLT [53]. Thus, the relationship between fibrinogen level and the development of AKI could be different between DDLT and LDLT. Further studies are required to confirm the predictive value of the fibrinogen level for AKI in DDLT patients. Fourth, the etiology of liver disease could have affected the results and generalizability of our findings. The indications for LDLT vary both between countries and between centers [54]. In addition, viral infection can result in renal injury [55, 56]. However, AKI is well known to be related to hepatic decompensation and portal hypertension in cirrhotic patients [45]. Further studies are required to elucidate the relation between etiology and development of AKI in patients with low fibrinogen levels.

## Conclusions

AKI is a common postoperative complication in patients undergoing LT, and is associated with increased mortality and poor graft survival. The risk factors for the development of AKI need to be assessed before surgery, and patients should be monitored carefully during the preoperative period. Our study suggests that a low fibrinogen level is a promising marker for the development of AKI, and could provide useful information for understanding the patient’s condition. Therefore, our study could facilitate the early identification of vulnerability to AKI after LDLT.

## Supporting information

S1 Table Incidence of AKI in PS-matched patients according to postoperative day. (DOCX)

S2 Table Association between the fibrinogen level and postoperative development of AKI after adjusting for preoperative factors. (DOCX)

## Abbreviations

AKI: acute kidney injury
BMI: body mass index
CKD: chronic kidney disease
CRP: C-reactive protein
DDLT: deceased donor liver transplantation
DM: diabetes mellitus
FFP: fresh frozen plasma
ICU: intensive care unit
IL: interleukin
INR: international normalized ratio
IQR: interquartile
LDLT: living donor liver transplantation
LT: liver transplantation
MELD score: model for end-stage liver disease score
POD: postoperative day
PRBC: packed red blood cells
PRS: postreperfusion syndrome
PS: propensity score
SDP: single donor platelet
sCr: serum creatinine
WBC: white blood cell
