# Supplementary material for: Predictive utility of fibrinogen in acute kidney injury in living donor liver transplantation: A propensity score-matching analysis
Source: PLoS One. 2021 Jun 4;16(6):e0252715. doi: 10.1371/journal.pone.0252715 (PMC8177619; doi:10.1371/journal.pone.0252715)
Supplement: S1 Table — (DOCX) [file pone.0252715.s001.docx]

**S1 Table. Incidence of AKI in PS-matched patients according to postoperative day.**

| **Group** | **incidence of AKI** | ***p*** |
| --- | --- | --- |
| **Postoperative day** | **76** | 0.005 |
| POD 1 | 46 (60.5%) |  |
| POD 2 | 25 (32.9%) |  |
| POD 3 | 2 (2.6%) |  |
| POD 4 | 2 (1.3%) |  |
| POD 5 | 1 (0%) |  |
| POD 6 | 0 (0%) |  |
| POD 7 | 0 (0%) |  |

**Abbreviations:** AKI, acute kidney injury; POD, postoperative day

**NOTE:** Values are expressed as numbers (proportions).
