# Supplementary material for: Predictive utility of fibrinogen in acute kidney injury in living donor liver transplantation: A propensity score-matching analysis
Source: PLoS One. 2021 Jun 4;16(6):e0252715. doi: 10.1371/journal.pone.0252715 (PMC8177619; doi:10.1371/journal.pone.0252715)
Supplement: S2 Table — (DOCX) [file pone.0252715.s002.docx]

**S2 Table. Association between the fibrinogen level and postoperative development of AKI after adjusting for preoperative factors.**

|  | ***ß*** | **Odds ratio** | **95% CI** | ***p*** |
| --- | --- | --- | --- | --- |
| **In the entire study population (n=615)** |  |  |  |  |
| Fibrinogen (mg/dL; continuous) adjusted for preoperative factors and PS | –0.005 | 0.995 | 0.992–0.997 | <0.001 |
| Low fibrinogen (vs. normal fibrinogen)† adjusted for preoperative factors †† and PS | 0.827 | 2.286 | 1.527–3.424 | <0.001 |
| **In the PS-matched study population (n=336)** |  |  |  |  |
| Fibrinogen (mg/dL; continuous) adjusted for preoperative factors and PS | –0.006 | 0.994 | 0.990–0.998 | 0.006 |
| Low fibrinogen (vs. normal fibrinogen)† adjusted for preoperative factors †† and PS | 0.766 | 2.150 | 1.267–3.651 | 0.005 |

**Abbreviations:** AKI, acute kidney injury; PS, propensity score

^†^(*vs*. reference values)

††(preoperative factors include body mass index, presence of diabetes mellitus, model for end-stage liver disease score, and albumin level)
